# Supplementary material for: Content-rich biological network constructed by mining PubMed abstracts
Source: BMC Bioinformatics. 2004 Oct 8;5:147. doi: 10.1186/1471-2105-5-147 (PMC528731; doi:10.1186/1471-2105-5-147)
Supplement: Additional File 5 — The original Chilibot query results of the term "long-term potentiation (LTP)" and 22 other terms, limiting the latest references analyzed to the years 1990, 1995, 2000, and 2004. [file 1471-2105-5-147-S5.bz2 › chilibotAdditionalFile5/ltp2004/html/CREB_PKA.html]

 


 **CREB** and **PKA** 
  
Found 312 abstracts in PubMed,  **30 abstracts were retrieved and analyzed**.  


---

 Search Google  |
 PDF files only 
|  EDU domain only 

---

**Interactive relationship** (e.g. stimulation, inhibition, etc)

- These results suggest strongly that  **PKA**  simultaneously plays two different roles in neuronal differentiation inhibition of neuritogenesis and stimulation of synaptogenesis, via  **CREB**  mediated gene expression.  Ref: 14648587 J Neurosci Res, 2003
- Inhibition of cAMP dependent protein kinase  **PKA**  using H89 N 2 p bromocinnamyl amino ethyl 5 isoquinolinesulfonamide blocked phosphorylation of the cAMP response element binding protein  **CREB**  and p38, but did not decrease NECA induced ERK1 2 phosphorylation.  Ref: 14516797 Exp Cell Res, 2003
- Inhibition of  **PKA**  impaired context and tone dependent fear conditioning and significantly reduced the phosphorylation of Elk 1, p90Rsk 1, Erk 1 2, and  **CREB** .  Ref: 14739006 Behav Brain Res, 2004
- Within the  **PKA**  pathway, SAD inhibited the activity of the catalytic subunit of  **PKA**  and its migration into the nucleus, elevated phosphorylated cyclic AMP cAMP response element CRE binding protein pCREB level, and reduced the binding of  **CREB**  to CRE.  Ref: 14761683 Toxicol Appl Pharmacol, 2004
- In addition, a specific  **PKA**  inhibitor that blocked the phosphorylation of  **CREB**  and c Jun by luteolin partially reversed the inhibitory effect of luteolin on NF kappa B.CBP complex formation and NF kappa B transcriptional activity by LPS.  Ref: 12963482 Biochem Pharmacol, 2003
- The EPAC specific cAMP analogue 8CPT 2Me cAMP activates Rap1 in B CLL cells, but, unlike rolipram forskolin or 8 Br cAMP, does not induce  **PKA**  activation, as judged by phosphorylation of the transcription factor  **CREB** .  Ref: 14615375 BloodBlood, 2003
- The protein kinase A  **PKA**  inhibitor H89 completely blocks the TCDD dependent effect on C EBP b promoter activity, indicating that TCDD activates  **CREB**  binding via a cAMP  **PKA**  pathway which is supported by the increased cAMP level and  **PKA**  activity observed after TCDD treatment.  Ref: 14684744 J Biol ChemJ Biol Chem, 2003
- Erk 1 2, Elk 1, and  **CREB**  were identified as common targets of  **PKA** , PKC, and CaMK II during memory consolidation the time window and sequence of their phosphorylation was specific for the individual kinase.  Ref: 14739006 Behav Brain Res, 2004
- During precipitated withdrawal, D1 receptor mediated  **CREB**  phosphorylation was dependent on cAMP dependent protein kinase  **PKA** .  Ref: 12969258 J Neurochem, 2003
- cAMP elevating agents did not inhibit nuclear translocation and DNA binding of SMAD3 4 complexes, but abolished the interactions of SMAD3 with the transcription coactivators  **CREB**  binding protein CBP and p300 in a  **PKA**  dependent manner.  Ref: 14654784 Oncogene, 2003
- The mechanism through which cAMP  **PKA**  signaling modulates the development of tolerance remains to be elucidated but may involve changes in phospho  **CREB**  expression.  Ref: 12966313 Alcohol Clin Exp Res, 2003
- Immunoblot analyses revealed that  **PKA**  activity mediates phosphorylation of a gene transcription factor, cAMP response element binding protein  **CREB** .  Ref: 14648587 J Neurosci Res, 2003
- The activity of  **CREB**  transcription factor is regulated through phosphorylation of Ser 133 by one of several protein kinases e.g. protein kinase A  **PKA** , p90 ribosomal S6 kinases RSKs and the related kinase, MSK1.  Ref: 14741741 Biochim Biophys Acta, 2004

**Parallel relationship** (e.g. studied together, co-existance, homology, etc.)

- **PKA**   **CREB**  gain of function enhanced BMP stimulation of this reporter, while loss of  **CREB**  function diminished transcriptional activity.  Ref: 14755548 J Cell Physiol, 2004
- To analyze the downstream consequences of ERK and  **PKA**  signaling, we studied the action of visual stimulation on transcription of genes controlled by  **CREB**  in transgenic mice carrying the LacZ reporter gene under the control of the CRE cAMP response element promoter.  Ref: 12904462 J Neurosci, 2003
- DIM stimulated MAPK activity and induced phosphorylation of the endogenous  **PKA**  target, cAMP response element binding protein  **CREB** , in a  **PKA**  dependent manner.  Ref: 14645498 Mol Endocrinol, 2004
- These findings are the first to demonstrate a cooperative association between  **CREB**  and BMP regulated Smads in cells from vertebrates and demonstrate that promoter specific rather than generalized interactions between  **PKA**   **CREB**  and BMP signaling regulate gene expression in chondrocytes.  Ref: 14755548 J Cell Physiol, 2004
- These results suggest that suppression of TGF beta SMAD signaling and resulting gene transactivation by cAMP inducing agents occurs via  **PKA**  dependent,  **CREB**  mediated, disruption of SMAD CBP p300 complexes.  Ref: 14654784 Oncogene, 2003
- Furthermore, we reported that normalization of  **CREB**  phosphorylation by infusing protein kinase A  **PKA**  activator into the central amygdala prevents anxiety like effects in rats during ethanol withdrawal.  Ref: 14706555 Peptides, 2003
- Then, the enzyme activity and protein levels of  **PKA**  Calpha subunit and phopsho cAMP responsive element binding  **CREB**  were assayed in lymphoblasts derived from 12 BAD and 12 control CT subjects and cultured for 24 h in the presence of cAMP analog drugs.  Ref: 14735135 NeuropsychopharmacologyNeuropsychopharmacology, 2003
- No changes in phospho  **CREB**  expression were detected in these brain regions in mice with heterozygous inactivation of the Gnas gene. The results show that cAMP  **PKA**  signal transduction modulates sensitivity to sedative and hypothermic effects of ethanol.  Ref: 12966313 Alcohol Clin Exp Res, 2003
- The early cAMP increase was effective in activating cAMP dependent protein kinase  **PKA**  and increasing phosphorylation of  **CREB**  transcription factor.  Ref: 14506285 J Biol Chem, 2003
- The new synapses are enriched in NMDA receptor and it was found that their generation involves activation of NMDA receptors,  **PKA**  and  **CREB** .  Ref: 14560699 Neurol Neurochir Pol, 2003
- Cotransfection experiments with  **CREB**  and  **PKA**  expression plasmids further supported our conclusions that the TCDD dependent effect on C EBP b transcription is mediated via  **PKA**  dependent  **CREB**  activation.  Ref: 14684744 J Biol ChemJ Biol Chem, 2003
- We investigated the involvement of hippocampal protein kinase A  **PKA** , protein kinase C PKC, calcium calmodulin dependent protein kinases CaMK II, and mitogen activated extracellular signal regulated kinases 1 and 2 Mek 1 2 in the phosphorylation of their downstream targets extracellular signal regulated kinases 1 and 2 Erk 1 2, cAMP response element binding protein  **CREB** , Elk 1, and p90 ribosomal S6 kinase 1 p90Rsk 1.  Ref: 14739006 Behav Brain Res, 2004
- These results demonstrate that DIM is a mechanistically novel activator of the ER that requires  **PKA**  dependent phosphorylation of  **CREB** .  Ref: 14645498 Mol Endocrinol, 2004
- Inversely, coexpression of either an active  **PKA**  catalytic subunit or that of the cAMP response element CRE binding protein  **CREB**  blocked SMAD driven gene transactivation.  Ref: 14654784 Oncogene, 2003
- Since adenosine receptor occupancy has been associated with activation of the cAMP  **PKA**  system as well as of p38 MAPK and p42 44 MAPK, all of which can activate the  **CREB**  transcription factor system, we hypothesized that adenosine would activate  **CREB**  in macrophages.  Ref: 14651954 Biochem Biophys Res Commun, 2003
- Here we investigated whether normalization of  **CREB**  phosphorylation by infusing  **PKA**  activator Sp cAMP into the central amygdala also normalizes the expression of NPY during ethanol withdrawal.  Ref: 14706555 Peptides, 2003
- Recently, it has been shown that ERK extracellular signal regulated kinase 1 2, protein kinase A  **PKA** , and  **CREB**  cAMP response element binding protein are crucial factors for experience dependent development of the visual cortex, but very little is known about the role of visual experience in their activation.  Ref: 12904462 J Neurosci, 2003
- PTHrP inhibits maturation through stimulation of  **PKA**   **CREB**  signaling while the bone morphogenetic proteins BMPs stimulate maturation through Smad mediated signaling.  Ref: 14755548 J Cell Physiol, 2004
- Via  **PKA** , PKC and p38 MAPK dependent signals, dBcAMP also induced inhibitory subunit of NF kappaB IkappaB degradation, IkappaB kinase IKK activation, nuclear translocation of NF kappaB subunit p65 and its association with the  **CREB**  binding protein CBP.  Ref: 14751542 Cell Signal, 2004
- Further, we have also clearly demonstrated that cAMP stimulates melanogenesis through the activation of the  **PKA**   **CREB**  pathway leading to the stimulation of Mitf expression.  Ref: 12950747 Pigment Cell Res, 2003
